# Age‐related risk of household transmission of COVID‐19 in Singapore

**DOI:** 10.1111/irv.12809

**Published:** 2020-09-29

**Authors:** Rachael Pung, Minah Park, Alex R. Cook, Vernon J. Lee

**Affiliations:** ^1^ Ministry of Health Singapore City Singapore; ^2^ Saw Swee Hock School of Public Health National University of Singapore Singapore City Singapore; ^3^ National University Health System Singapore City Singapore

## Abstract

Prior to the implementation of social distancing measures, we monitored the close family contacts of the first 400 cases of COVID‐19 in Singapore for SARS‐CoV‐2 infection to determine the risk of infection with age. Adjusting for gender and household size, the risk of COVID‐19 infection in household contacts was found to increase with age.

## 1 INTRODUCTION

Determining the transmission of SARS‐CoV‐2 in households is crucial for understanding the heterogeneities in the age‐related risk of infection, as the exposures will likely be high given the strong familial interactions. The outcome from this analysis is important to guide containment measures for the ongoing pandemic such as school closures and healthcare resource allocation.

## 2 METHODOLOGY

Close household contacts of a laboratory‐confirmed COVID‐19‐infected case in Singapore, a city‐state in South‐East Asia with a population of 5.7 million, were contact traced and interviewed for demographic characteristics. We limit our analysis to contacts of the first 400 COVID‐19‐infected cases in Singapore, that is those identified before 21 March 2020 as the number of cases in the population with unidentified source of infection was less than 40 at that point and the likelihood of household cases being exposed to the population was low. This is also prior to the tightening of precautionary measures introduced by the government to minimise the spread of COVID‐19^1^, ^2^ that may have modified the risk of acquiring infection in the home.

We tested household contacts for SARS‐CoV‐2 when (a) they exhibit recent or active symptoms compatible with COVID‐19 or (b) they are aged 12 and below (with effect from March 2020), regardless of their symptoms, as this age group may not report symptoms. Those testing negative and other well household contacts were quarantined at home or in government quarantine facilities and monitored daily for development of symptoms for 14 days from their last exposure to a case.

Multivariate logistic‐regression model was used to examine the relationship between infection outcome of a household contact and age, adjusted by the duration of exposure. Exposure between each case and household contact is assumed to be independent and measured from the case's symptom onset to last date of exposure with the household contact or to symptoms onset in the infected household contact, whichever comes first. The duration of exposure for each household contact is the sum of their exposures to all cases in the household (for household contacts who were infected, the duration of exposure is the sum of their exposure to all cases preceding their infection). Sensitivity analysis was performed to adjust for potential demographic confounders such as gender and household size. Household contacts with the same exposure outside of the household as the household index case and cases who practice self‐isolation from their household contacts were omitted from the analysis.

We computed the 95% confidence intervals (CI) for secondary clinical attack rate (SCAR) among household contacts.^3^ One‐way ANOVA was used to evaluate the difference in the mean secondary infection rate in household of different sizes. We also report the serial interval—defined as time from symptom onset in an infector to symptom onset in an infectee, using data from households with only one newly infected household contact (ie no tertiary transmission to confound the serial interval analysis). All analysis was done using R version 3.5.1.^4^

## 3 RESULTS

Of the 400 cases identified before 21 March 2020, 46 cases and their households were omitted due to common exposure between cases and household contacts or cases reported to self‐isolate from their household contacts. Of the remaining cases, 34 had no household contacts, 277 were primary or co‐primary cases in their household and 43 household contacts were tested positive for SARS‐CoV‐2. A total of 265 households and 875 household contacts were identified, and the overall median household size was 4 (IQR 3‐6).

**FIGURE 1:**
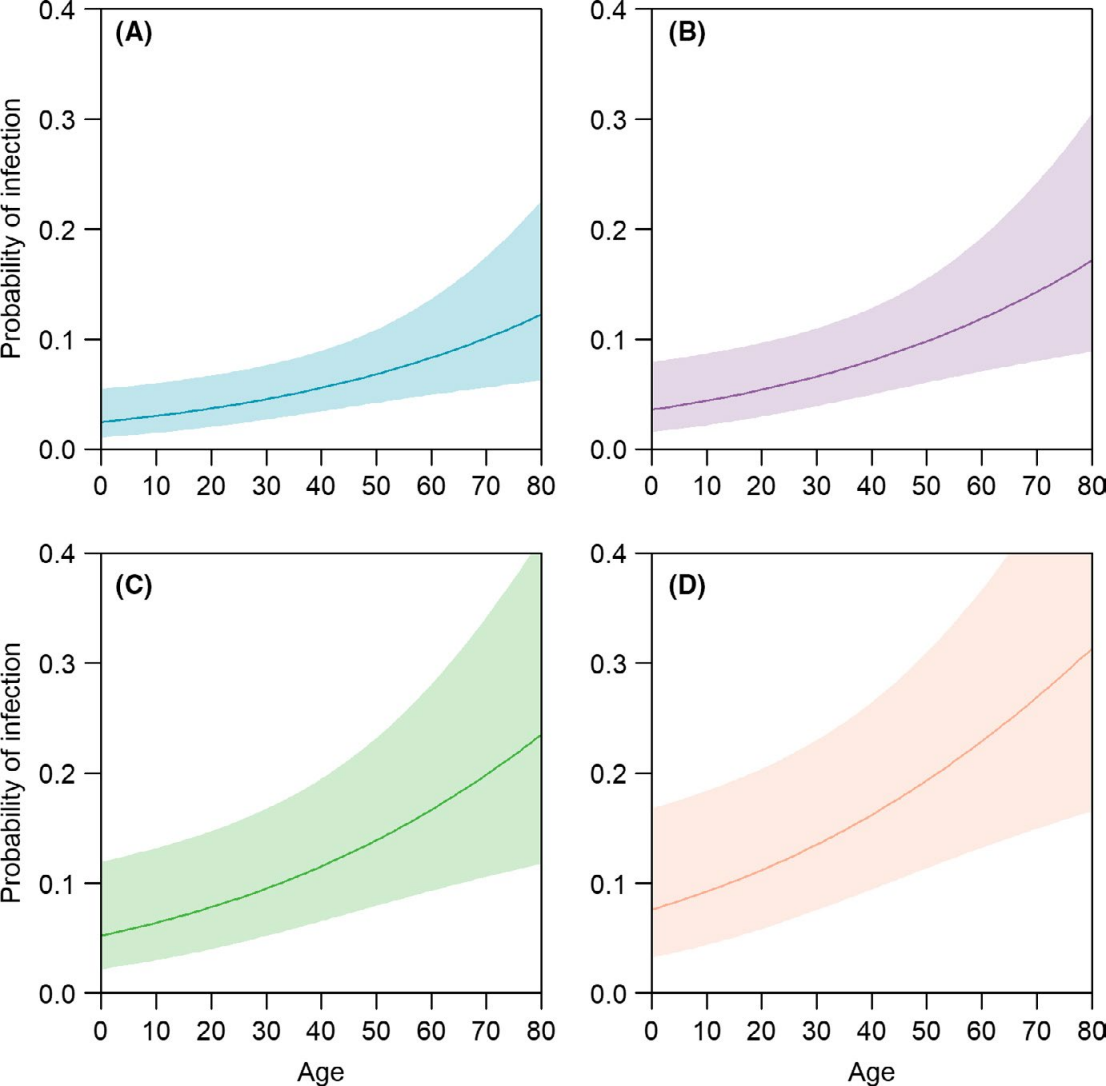
Mean probability of infection by adjusted by age, gender and household size (line) and 95% confidence interval (shaded area), (A) adjusted for duration of exposure of 5 d (turquoise), (B) 10 days (purple), (C) 15 d (green) and (D) 20 d (orange)

After adjusting for the duration of exposure, risk of infection was estimated to increase with age (Figure 1). This association was strengthened after further adjustment by gender and number of household contacts (Appendix S1). The mean SCAR was 3.8% (95% CI 2.1‐6.9) and did not vary significantly with household size. The mean serial interval from 36 pairs of cases and the earliest infected household contacts was 6.1 days (IQR 3.8‐8.0).

## 4 DISCUSSION

Our study suggests that the risk of COVID‐19 infection in the household setting increases with age. This finding corroborates with other studies on the demographic characteristics of COVID‐19‐infected cases in the population.^5^ It also supports local observations from two pre‐school outbreaks, one involving 16 adult staff who acquired infection within the pre‐school through one of the staffs who continued to work when ill, while none of the 77 out of 112 students who were close contacts and whose parents consented their being tested were positive for SARS‐CoV‐2. In another pre‐school outbreak involving 2 adult staff and a 2‐year old pre‐schooler taught by one of the infected staffs, 17 (out of 30) students were close contacts and were tested, but none were positive for SARS‐CoV‐2 (unpublished data). These data suggest that children are less likely to be infected and hence not the major drivers of transmission unlike in other respiratory diseases such as influenza and RSV.^6^ As such, it appears inadvisable that school closures be the main community‐based measure taken to reduce community transmission. However, school closures have been rolled out in many countries as part of physical distancing measures. This could serve to minimise interactions between adult and elderly workers in schools and to ensure that parents remain home to care for their children.

Outcomes of this study were not affected by censoring of the data as contacts were monitored until the end of their quarantine period. The mean SCAR of 3.8% was similar to 2009 influenza A H1N1 pandemic^7^ but lower than that of seasonal influenza.^8^ However, the lack of children index cases (zero case) limits our ability to evaluate infectiousness of COVID‐19 by age group.

While symptomatic contacts and asymptomatic children aged 12 and below were referred for testing, respiratory samples were not collected from other healthy household contacts. Thus, we were unable to ascertain the true proportion of asymptomatic cases and hence secondary infection rate. Furthermore, we did not interview the household contacts for clinical risk factors for COVID‐19 and this could confound the analysis. With ongoing clinical trials for post‐exposure prophylaxis of SARS‐CoV‐2 infection, the findings from the current study would serve as a comparison to other household studies with the administration of such intervention in the future.

## AUTHOR CONTRIBUTION

**Rachael Pung:** Conceptualization (equal); Formal analysis (lead); Investigation (lead); Methodology (lead); Writing‐original draft (lead); Writing‐review & editing (lead). **Minah Park:** Conceptualization (equal); Investigation (supporting); Methodology (supporting); Writing‐review & editing (supporting). **Alex R. Cook:** Conceptualization (equal); Methodology (supporting); Supervision (equal); Writing‐original draft (supporting); Writing‐review & editing (supporting). **Vernon J. Lee:** Conceptualization (equal); Supervision (equal); Writing‐original draft (supporting); Writing‐review & editing (supporting).

## Supporting information

Appendix S1
