# Supplementary material for: Age‐related risk of household transmission of COVID‐19 in Singapore
Source: Influenza Other Respir Viruses. 2020 Sep 29;15(2):206–8. doi: 10.1111/irv.12809 (PMC7646651; doi:10.1111/irv.12809)
Supplement: Supplementary file 1 — Appendix S1 [file IRV-15-206-s001.docx]

**Supplementary Info**


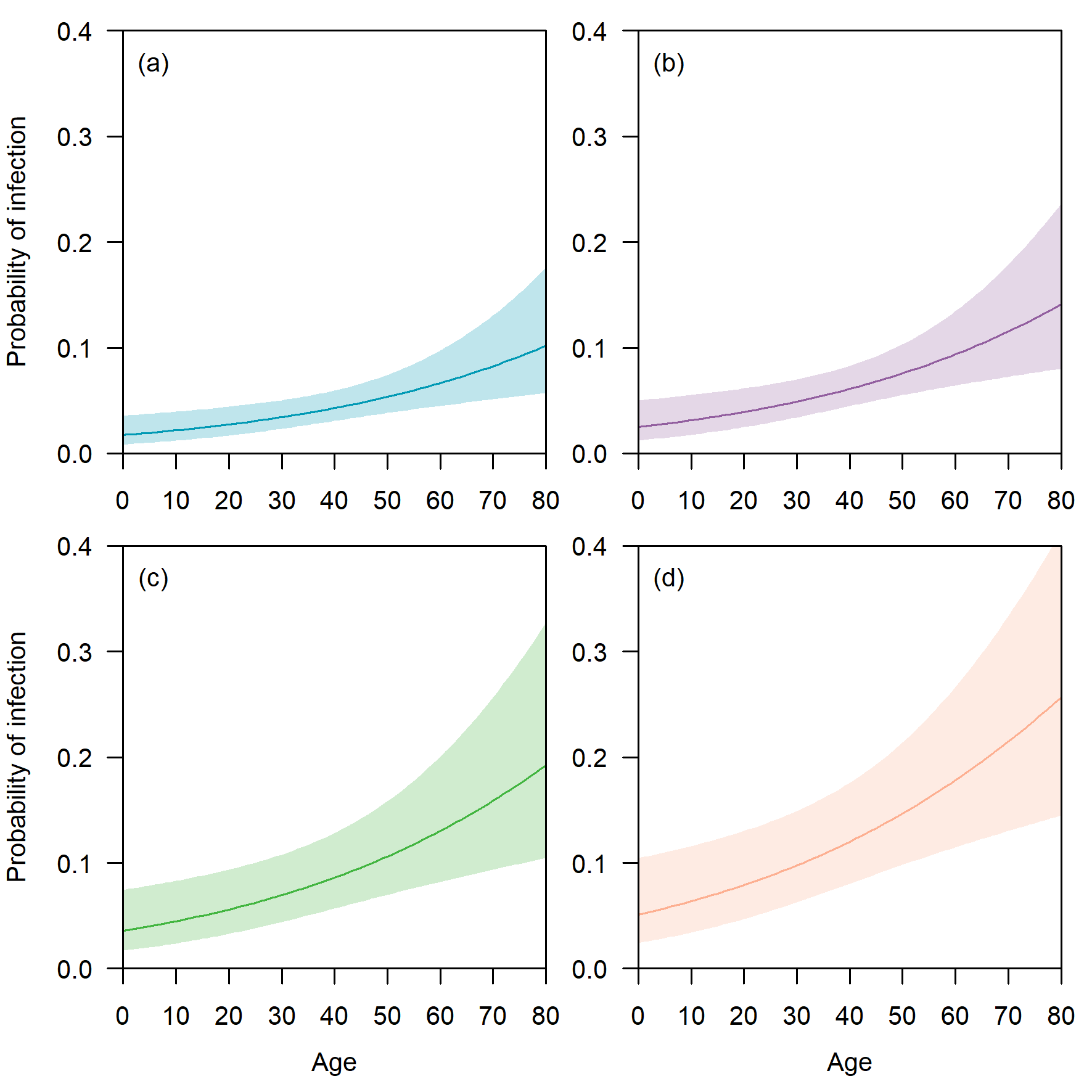


Supplementary Figure 1 Mean probability of infection adjusted by age only (line) and 95% confidence interval (shaded area), (a) adjusted for duration of exposure of 5 days (turquoise), (b) 10 days (purple), (c) 15 days (green) and (d) 20 days (orange)

Supplementary Table 1 Mean risk of infection (95% CI) adjusted by age only

| Age | 5d Exposure | 10d Exposure | 15d Exposure | 20d Exposure |
| --- | --- | --- | --- | --- |
| 10 | 2.1% (1.2%, 3.9%) | 3.1% (1.7%, 5.5%) | 4.4% (2.3%, 8.2%) | 6.2% (3.3%, 11.5%) |
| 20 | 2.7% (1.6%, 4.4%) | 3.8% (2.4%, 6.1%) | 5.5% (3.2%, 9.2%) | 7.7% (4.6%, 12.9%) |
| 30 | 3.4% (2.3%, 5.0%) | 4.8% (3.3%, 6.9%) | 6.8% (4.3%, 10.6%) | 9.6% (6.1%, 14.7%) |
| 40 | 4.2% (3.0%, 5.8%) | 6.0% (4.4%, 8.1%) | 8.4% (5.6%, 12.6%) | 11.8% (7.9%, 17.3%) |
| 50 | 5.2% (3.8%, 7.3%) | 7.4% (5.4%, 10.1%) | 10.4% (6.9%, 15.5%) | 14.4% (9.6%, 21.0%) |
| 60 | 6.5% (4.4%, 9.5%) | 9.2% (6.3%, 13.1%) | 12.8% (8.1%, 19.6%) | 17.5% (11.3%, 26.1%) |
| 70 | 8.1% (5.1%, 12.7%) | 11.3% (7.2%, 17.4%) | 15.6% (9.2%, 25.0%) | 21.1% (12.9%, 32.6%) |
| 80 | 10.0% (5.6%, 17.1%) | 13.8% (7.9%, 23.0%) | 18.9% (10.3%, 31.9%) | 25.2% (14.3%, 40.5%) |

Supplementary Table 2 Mean risk of infection (95% CI) adjusted by age, gender and household size

| Age | 5d Exposure | 10d Exposure | 15d Exposure | 20d Exposure |
| --- | --- | --- | --- | --- |
| 10 | 3.0% (1.4%, 6.0%) | 4.3% (2.1%, 8.6%) | 6.3% (2.9%, 13.1%) | 9.1% (4.3%, 18.2%) |
| 20 | 3.6% (2.0%, 6.6%) | 5.3% (2.9%, 9.6%) | 7.7% (3.9%, 14.6%) | 11.0% (5.7%, 20.2%) |
| 30 | 4.5% (2.6%, 7.5%) | 6.5% (3.8%, 10.8%) | 9.3% (5.1%, 16.5%) | 13.3% (7.4%, 22.7%) |
| 40 | 5.5% (3.4%, 8.8%) | 7.9% (4.9%, 12.6%) | 11.3% (6.4%, 19.2%) | 15.9% (9.2%, 26.1%) |
| 50 | 6.7% (4.1%, 10.7%) | 9.6% (5.9%, 15.2%) | 13.6% (7.8%, 22.8%) | 19.0% (11.2%, 30.5%) |
| 60 | 8.2% (4.9%, 13.3%) | 11.7% (7.0%, 18.8%) | 16.4% (9.2%, 27.5%) | 22.5% (13.0%, 36.1%) |
| 70 | 9.9% (5.6%, 17.1%) | 14.0% (7.9%, 23.7%) | 19.5% (10.5%, 33.5%) | 26.5% (14.8%, 42.8%) |
| 80 | 12.0% (6.2%, 22.0%) | 16.8% (8.8%, 29.8%) | 23.1% (11.7%, 40.6%) | 30.9% (16.4%, 50.4%) |
